# Pathological consequences of *MICU1* mutations on mitochondrial calcium signalling and bioenergetics^☆^

**DOI:** 10.1016/j.bbamcr.2017.01.015

**Published:** 2017-06

**Authors:** Gauri Bhosale, Jenny A. Sharpe, Amanda Koh, Antonina Kouli, Gyorgy Szabadkai, Michael R. Duchen

**Affiliations:** a Department of Cell and Developmental Biology, University College London, Gower Street, London WC1E 6BT, United Kingdom; b Department of Biomedical Sciences, University of Padua, 35131 Padua, Italy

**Keywords:** Mitochondria, Calcium, MICU1, PDH

## Abstract

Loss of function mutations of the protein MICU1, a regulator of mitochondrial Ca^2 +^ uptake, cause a neuronal and muscular disorder characterised by impaired cognition, muscle weakness and an extrapyramidal motor disorder. We have shown previously that MICU1 mutations cause increased resting mitochondrial Ca^2+^ concentration ([Ca^2 +^]_m_). We now explore the functional consequences of *MICU1* mutations in patient derived fibroblasts in order to clarify the underlying pathophysiology of this disorder. We propose that deregulation of mitochondrial Ca^2+^ uptake through loss of MICU1 raises resting [Ca^2+^]_m_, initiating a futile Ca^2+^ cycle, whereby continuous mitochondrial Ca^2+^ influx is balanced by Ca^2+^ efflux through the sodium calcium exchanger (NLCX_m_). Thus, inhibition of NCLX_m_ by CGP-37157 caused rapid mitochondrial Ca^2+^ accumulation in patient but not control cells. We suggest that increased NCLX activity will increase sodium/proton exchange, potentially undermining oxidative phosphorylation, although this is balanced by dephosphorylation and activation of pyruvate dehydrogenase (PDH) in response to the increased [Ca^2+^]_m_. Consistent with this model, while ATP content in patient derived or control fibroblasts was not different, ATP increased significantly in response to CGP-37157 in the patient but not the control cells. In addition, EMRE expression levels were altered in MICU1 patient cells compared to the controls. The MICU1 mutations were associated with mitochondrial fragmentation which we show is related to altered DRP1 phosphorylation. Thus, MICU1 serves as a signal–noise discriminator in mitochondrial calcium signalling, limiting the energetic costs of mitochondrial Ca^2+^ signalling which may undermine oxidative phosphorylation, especially in tissues with highly dynamic energetic demands. This article is part of a Special Issue entitled: ECS Meeting edited by Claus Heizmann, Joachim Krebs and Jacques Haiech.

## Highlights

• Loss of MICU1 protein expression in human fibroblasts increases resting mitochondrial calcium concentration ([Ca^2+^]_m_).
• The increased mitochondrial Ca^2+^ uptake causes a futile Ca^2+^ cycle in MICU1 deficient cells.
• Increased [Ca^2+^]_m_activates pyruvate dehydrogenase (PDH) by activating PDH phosphatase, consequently dephosphorylating PDH.
• Loss of *MICU1* leads to modifications of the MCU complex composition and mitochondrial fragmentation.

## 1 Introduction

Calcium signalling is fundamental to much of cell physiology, as a rise in cytosolic calcium ion concentration ([Ca^2+^]_c_) drives an astonishing array of physiological processes. These include contraction in skeletal, cardiac and smooth muscle, secretion from all cell types, while Ca^2+^ signals play key roles in learning and memory, in cell migration, and triggering the earliest phases of development following fertilisation of the oocyte. It has been clear since the pioneering work of Lehninger, Attardi, Carafoli, Deluca and Crompton that mitochondria have a huge capacity to accumulate calcium ions (Ca^2+^) [1], [2], [3], [4], [5]. The last two decades have seen the widespread recognition that all physiological calcium signals so far studied are associated with the accumulation of Ca^2+^ into mitochondria mediated by mitochondrial Ca^2+^ uptake pathways [6].

The accumulation of Ca^2+^ by mitochondria underpins a complex reciprocal dialogue with the Ca^2+^ signalling machinery that operates on many levels. Thus, the spatial buffering of Ca^2+^ by mitochondria serves to regulate the spatiotemporal patterning of Ca^2+^ signals [7], which may have a profound impact on downstream Ca^2+^ dependent signalling pathways. At the same time, a rise in [Ca^2+^]_c_ and an increase in matrix Ca^2+^ concentration ([Ca^2 +^]_m_) both have metabolic consequences. A rise in [Ca^2+^]_c_ will drive an increase in ATP consumption, but simultaneously stimulates the malate-aspartate shuttle, ARALAR, driving an increase in intramitochondrial NADH that stimulates respiration and increases the rate of ATP synthesis [8]. This is amplified by the impact of a rise in [Ca^2+^]_m_, which stimulates the activity of the three rate limiting enzymes of the TCA cycle, each of which is modulated by Ca^2+^, again increasing the rate at which reduced NADH is generated by the cycle, and so driving an increased rate of ATP synthesis [9]. This increased activity is balanced and supported by stimulation of the ATP synthase itself, perhaps less clearly characterised [10], [11], [12], [13], [14]. Thus, the dialogue between mitochondria and Ca^2+^ signalling reflects a simple and elegant mechanism that serves to balance an increased rate of ATP provision to match the increased demand that inevitably accompanies the processes driven by the Ca^2+^ signal – an increase in work through activation of contraction, secretion, migration, or gene expression.

Mitochondrial Ca^2+^ uptake also drives cell death under conditions of cellular Ca^2+^ overload, as supraphysiological mitochondrial Ca^2+^ accumulation can trigger opening of a large conductance pore in the inner mitochondrial membrane, the mitochondrial permeability transition pore (mPTP) [15], [16], [17], especially when coincident with oxidative stress. Ca^2+^ induced cell death has been most extensively characterised in ischaemia reperfusion injury in the heart [18], [19], but probably also plays roles in in neurodegenerative disorders such as ALS, Alzheimer's disease and Parkinson's disease [20], possibly in demyelinating disease (multiple sclerosis), in pancreatitis, in several forms of muscular dystrophy and myopathy [21] and in pathological changes associated with diabetes [22], [23].

Ca^2+^ homeostasis within the mitochondrial matrix is maintained through Ca^2+^ uptake and efflux pathways. The primary mechanism for Ca^2+^ efflux that normally maintains a low matrix Ca^2+^ concentration ([Ca^2+^]_m_) is the Na^+^/Ca^2+^ exchanger, recently identified as NCLX [24]. While the capacity of energised mitochondria to accumulate Ca^2+^ was first observed in the 1960s, the molecular identity of the channel that mediates Ca^2+^ import into mitochondria was identified only recently as the well conserved mitochondrial Ca^2+^ uniporter (MCU) [25], [26], a ruthenium-red sensitive channel in the inner mitochondrial membrane (IMM). The MCU consists of two highly conserved transmembrane domains connected by the DIME motif, which are predicted to oligomerise and form a tetrameric gated ion channel [27]. Knockout or silencing of the MCU in most mouse strains is embryonically lethal, but viable knockouts have been generated in an outbred strain [28]. In this model, MCU knockout severely reduces calcium uptake, but appears to have surprisingly little impact on mitochondrial bioenergetic function [25], [26], [29]. The global *MCU* knockout (*MCU* KO) mice are smaller than littermates, and show a reduced power and reduced activity on a treadmill but otherwise the phenotype is very mild. The conditional knockout in the heart shows a reduced capacity to respond to increased drive [30].

The MCU complex consists of the MCU in association with several proteins which are thought to play a regulatory role, and some of which show variation in expression in different tissues [27], [31]. This could be important in addressing the different metabolic demands of different tissues. MCU associated proteins include MCUb, MICU1, MICU2 and MICU3 and EMRE, and possibly some other proteins whose contribution remains a little more controversial (for example, MCUR1, SLC25A23) [32], [33]. Of these components, MICU1 and MICU2 (Mitochondrial Calcium Uptake 1) play significant roles in regulating calcium uptake. MICU1 has two highly conserved EF hand motifs, which confer sensitivity to cytosolic Ca^2+^ concentration [Ca^2+^]_c_ [34]. MICU2 also has Ca^2+^ sensing EF hands, which allow MICU2 to form dimers upon binding to Ca^2+^_._ The two proteins form a heterodimer via a disulphide bond and salt bridge [35]. MICU2 requires the expression of MICU1 for stability, as downregulation of MICU1 results in reduction of MICU2 levels, implying a strong correlation in expression levels [36]. It has been suggested that MICU2 inhibits MCU opening at low [Ca^2+^]_c_ levels, sensed by the EF hands in the intermembrane space [37]. Together, MICU1 and MICU2 establish a threshold [Ca^2+^]_c_ at which MCU will open, keeping MCU closed at low [Ca^2+^]_c_ – at concentrations found at rest in the cytosol - while the channel opens at [Ca^2+^]_c_ above 2–3 μM showing a cooperative increase in uptake as Ca^2+^ concentrations increase as described in the earliest studies of mitochondrial Ca^2+^ uptake [34], [37]. Another subunit of interest is EMRE, which has been shown to be essential for Ca^2+^ uptake through its interaction with both MCU and MICU1 [38]. EMRE seems to act as a scaffolding protein and is apparently required for the correct stoichiometric assembly of the complex. In addition, the role of EMRE as a mitochondrial matrix Ca^2+^ sensor has been identified in the complex regulation of the MCU [39]. Most recently, the importance of the turnover of EMRE by an m-AAA protease in preventing Ca^2+^-induced cell death was discovered [40].

The functional consequences of altered MICU1 expression were characterised initially by knockout or overexpression in cell lines [34], [41], [42], [43]. This was followed by the discovery of a number of children with a complex and previously unexplained disorder, including a mild cognitive deficit, neuromuscular weakness and a progressive extrapyramidal motor disorder, all of whom showed frame shift mutations of MICU1 [44]. Other features which have been previously associated with mitochondrial disease were also reported in some patients, including ataxia, microcephaly, opthalmoplegia, ptosis, optic atrophy and peripheral axonal neuropathy. More recently, two cousins with a homozygous deletion in *MICU1* were described, showing fatigue and lethargy amongst other symptoms [45]. Cellular assays on patient fibroblasts from both reports revealed altered mitochondrial Ca^2+^ uptake, resulting in increased mitochondrial Ca^2+^ load, but surprisingly, did not reveal significant consequences on oxidative phosphorylation or membrane potential, consistent with reports from studies in cell lines as well as *in vivo* [36], [43]. In addition, the mitochondrial network was more fragmented in cells from the patients compared to the controls. Unlike the *MCU* KO mouse, a whole body knockout of *MICU1* in the mouse has been reported to result in a high probability of perinatal lethality in two independent studies [46], [47]. Those mice that survived showed physical signs including ataxia and muscle weakness as well as biochemical abnormalities, recapitulating the pathology observed in the patients. The phenotype of these animals improved with age, apparently related to the downregulation of EMRE expression [47].

In the present study, we have further investigated the functional consequences of loss of *MICU1* expression in patient derived fibroblasts. Whole exome-sequencing of the patients reported by Logan et al. revealed a homozygous mutation at a splice acceptor site, c.1078-1G > C in *MICU1* in 11 of the 15 individuals and at a splice donor site, c.741 + 1G > A in the remaining 4 patients. Experiments were carried out in fibroblasts obtained from two of the patients with the c.1078/1G > C mutation (referred to below as ΔMICU1) and from age matched controls.

We here propose a mechanism which could explain a bioenergetic deficiency in the patients suggesting that increased Ca^2+^ uptake even at resting [Ca^2+^]_c_ is balanced by Ca^2+^ efflux through the NLCX, in turn driving increased activity of the sodium proton exchanger. We propose that, as a consequence, an increase in proton influx across the inner membrane would undermine the proton-motive force to drive ATP synthesis by the ATP synthase. We provide evidence for the existence of a futile mitochondrial Ca^2 +^ cycle in patient derived fibroblasts and to show that this cycle impairs ATP synthesis through oxidative phosphorylation.

## 2 Material and methods

### 2.1 Cell culture

Human fibroblasts were obtained from patient and control skin samples, from a previously published report [44]. The previous study was approved by the boards of the Leeds East and Great Ormond Street Hospital research ethics committees (references Leeds East 07/H1306/113 and GOSH 00/5802, respectively) and the institutional review board of the University of Leiden.

Cells were grown in Dulbecco's modified Eagle's medium (DMEM: 4.5 g/L glucose and pyruvate) containing 10% foetal bovine serum (FBS) and 1% penicillin/streptomycin (5000 U/mL, Gibco 15070-063) at 37 °C in 5% CO_2_. Where galactose conditions are indicated, fibroblasts were cultured in zero glucose DMEM with 4 mM L-glutamine (Invitrogen), 10% FBS (Invitrogen), 1 mM sodium pyruvate (Sigma), 0.1% *w*/*v* (5.5 mM) galactose (MP Biomedicals) and 1% PS (Invitrogen).

### 2.2 Western blotting

Following relevant drug treatment and/or media changes, fibroblasts were washed with PBS, scraped and centrifuged. Cell pellets were then lysed in RIPA buffer (150 mM NaCl, 0.5% sodium deoxycholic acid, 0.1% SDS, 1% Triton X-100, 50 mM Tris pH 8, 1 mM PMSF, PhosSTOP phosphatase inhibitors (Roche)) for 30 min on ice. Samples were subsequently centrifuged at 16,000 *g* at 4 °C and protein concentrations determined using Pierce BCA assay (Thermo Scientific).

When using antibodies for detecting phosphorylation, 15–40 μg of protein was boiled at 95 °C for 5 mins in NuPAGE 4X LDS sample buffer (Invitrogen) containing 5% β-mercapethanol. Proteins were separated using 4–12% NuPAGE Bis-Tris gels (Invitrogen) with MOPS running buffer (Invitrogen) and transferred onto nitrocellulose membranes using NuPAGE transfer buffer (Invitrogen) supplemented with 20% methanol. Membranes were washed with TBS-T and blocked with 3% BSA in TBS-T for 1 h at RT, followed by overnight incubation with primary antibody. Following 3 × 10 min washes in TBS-T, membranes were incubated with secondary antibody solution for 1–1.5 h at RT. After 3 × 5 min washes in TBS-T, the membranes were developed using Amersham ECL reagent (GE Healthcare) and imaged with a ChemiDoc system (BioRad). Densitometry analysis was carried out using ImageJ. For detecting EMRE expression levels, 50 μg of protein was boiled and processed as above, the only differences being the use of 12% NuPAGE Bis-Tris gels, polyvinylidene fluoride (PVDF) membrane and 5% milk in TBS-T as the blocking buffer.

Primary antibodies used were anti-C22orf32 Antibody (C-12) (rabbit, Santa Cruz, 1:100), anti-DRP1 (mouse, Abcam; 1:1000), anti-phospho DRP1 (Ser637) (rabbit, NEB; 1:1000), anti-LC3b (rabbit, Cell Signaling; 1:2000), anti-β-actin (mouse, Santa Cruz; 1:2000), anti-PDH E1α (mouse, Invitrogen; 1:1000) and anti-phospho PDH E1α (Ser293) (rabbit, Novus Biologicals; 1:1000). Secondary antibodies used were anti-mouse and anti-rabbit (both from Thermo Scientific and diluted 1:4000).

In order to assess the PDH state and minimise variability relating to substrate supply, protein samples were made approximately 2 h after refreshing the culture media. Culture plates were also snap frozen at − 80 °C before scraping to minimise any subsequent kinase and phosphatase activity. Western blotting for phosphorylated PDH (pPDH) was carried out first and then the same membrane was washed and re-probed overnight for total PDH. The proportion of pPDH was then expressed as average intensity of pPDH band / average intensity of total PDH band. For the DCA experiments, plated cells were treated with 0, 2.5 and 5 mM sodium dichloroacetate, 98% (DCA) (347795) concentrations (neutralised with 1 M NaOH) respectively for two hours prior to whole cell solubilisation.

### 2.3 Assessing mitochondrial Ca^2+^ dynamics

Cells were plated one day before imaging on 22-mm glass coverslips in 6-well plates (100,000 cells per well). Cells were incubated with 5 μM rhod-FF AM (Life Technologies, R23983) dyes supplemented with 0.002% pluronic acid, in recording buffer (Glucose, 10 mM; NaCl, 150 mM; KCl, 4.25 mM; NaH_2_PO_4_, 1.25 mM; NaHCO_3_, 4 mM; CaCl_2_, 1.2 mM; MgCl_2_, 1.2 mM; HEPES, 10 mM) at room temperature for 30 min. Prior to imaging, the dye was washed off and the solution was replaced with recording buffer. Images were acquired on a Zeiss 700 CLSM (excitation at 555 nm, emission at > 560 nm) using a 40 × objective and a 37 °C heated stage.

ImageJ was used for image analysis. ROIs were drawn around individual cells and a threshold was applied to the images to quantify the mean intensity of the signal localised to the mitochondria within each cell. Identical acquisition settings and threshold values were used in all experiments.

### 2.4 Oxygen consumption measurements

Oxygen consumption rates were measured using the Oroboros Oxygraph-2K (Oroboros Instruments, Innsbruck, Austria). The sensors in each chamber were calibrated in the respiration medium, prior to the experiment. Cells were trypsinised and resuspended at 1 million cells/mL in DMEM buffered with 20 mM HEPES, supplemented with 5.5 mM glucose (or 5.5 mM galactose for galactose-grown cells), 2 mM glutamine and 1 mM pyruvate. The cellular suspension was maintained at 37 °C and stirred at 750 rpm. Drug additions were performed using Hamilton syringes. Once resting rate had stabilised, 10 μM histamine was added to induce a Ca^2+^_−_ dependent rise in O_2_ consumption. After returning to resting rate, 2.5 μM oligomycin A was added to measure leak respiration, 1 μM FCCP to determine maximal oxidative capacity and 2.5 μM antimycin A to measure non-mitochondrial (background) O_2_ consumption. Data were acquired and analysed using the DatLab 5 software and each of the respiratory states was defined as the average value over a region of stabilised signal.

### 2.5 Measuring ATP levels in the cells

ATP was measured using the CellTiter-Glo Luminescent Cell Viability Assay (Promega, G7570) protocol. This protocol is based on the principle that bioluminescence is produced when the enzyme luciferase catalyses the reaction between luciferin (both present in the assay buffer) and ATP present in the cell. The luminescent signal is proportional to the amount of ATP present. Cells were seeded in white 96 well plates (20,000 cells/well) and the next day were incubated with one of the following treatments for 1 h at 37 °C: 1 μL/mL DMSO, 5 μM oligomycin A, 1 mM iodoacetic acid (IAA), 10 μM CGP-37157 or 10 mM 2-deoxyglucose (DG). The cells were allowed to equilibrate at RT before incubating with CellTiter-Glo® Reagent (Promega) for 10 min. Luminescence values proportional to ATP content were measured in a plate reader (Fluostar Optima, BMG Labtech) using a luminescence optic with 3 mm diameter light guide. Each condition was carried out with a minimum sample size of 3 per replicate.

### 2.6 Statistics

Statistical analysis was performed using Prism 6 (GraphPad software). Values are presented as mean ± standard error. N numbers indicate number of independent repeat experiments unless otherwise indicated. Where the means of two independent groups were being compared e.g. control group v ΔMICU1 group, two tail *t*-tests were applied to test significance to a *P* value of 0.05. Where the means of three or more independent groups were being compared, one-way analysis of variance (ANOVA) was used. When the effect of two different independent variables was being measured e.g. cell line and drug treatment, two-way ANOVA was used. When several comparisons between groups were being made, appropriate post hoc tests were used to correct for multiple testing.

## 3 Results

### 3.1 MICU1 mutations lead to a futile Ca^2+^ cycle

We have shown previously that fibroblasts from patients with mutations in *MICU1* showed an increase in resting [Ca^2+^]_m_, an increased rate of mitochondrial Ca^2+^ uptake in response to stimulation but no change in peak Ca^2+^ accumulation [44]. In trying to understand how and why such a change in Ca^2+^ homeostasis might give rise to the disorder seen in the children, we considered whether loss of MICU1 might increase susceptibility to Ca^2+^ induced cell death. Experiments using thapsigargin to promote Ca^2+^ induced cell death failed to show any significant difference between thapsigargin induced death in controls or in ΔMICU1 cells (data not shown), although rates of thapsigargin induced cell death in fibroblasts were very low.

Furthermore, it has been suggested that MICU1 knockout in cell models increases rates of ROS generation, which might contribute to increased cell death [48]. We therefore measured rates of ROS generation using dihydroethidium. We found no evidence of increased oxidative stress in the patient derived ΔMICU1 cells compared to controls (see supplementary methods and Fig. S1).

**Fig. 1:**
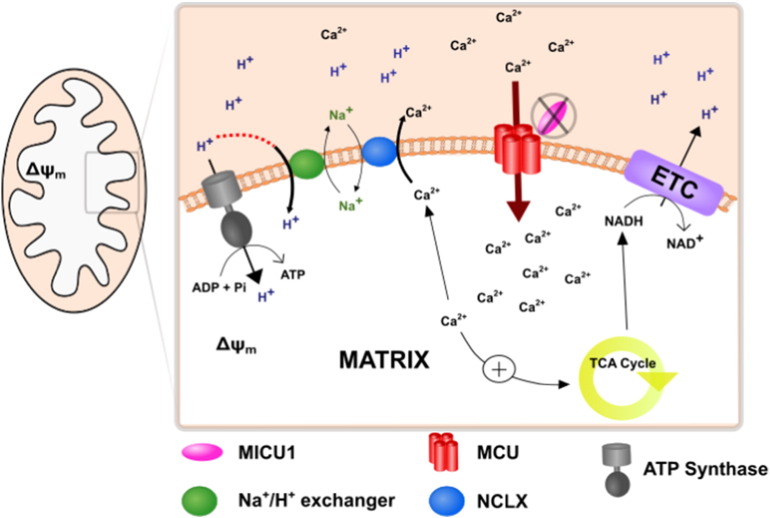
Schematic diagram to demonstrate the futile Ca^2+^ cycle established in the absence of MICU1, resulting in a deficit in ATP production.

We therefore wondered whether cellular energetics might be undermined by a futile mitochondrial Ca^2+^ cycle. In patients, increased mitochondrial Ca^2+^ uptake at rest through a loss of the threshold, ‘gatekeeping’ function of MICU1 raises [Ca^2+^]_m_. Increased matrix [Ca^2+^] will inevitably activate the NCLX, promoting Ca^2+^ efflux from the matrix. The concomitant increase in Na^+^ flux will in turn stimulate the Na^+^/H^+^ exchange (NHX), compromising the proton gradient available for ATP production (Fig. 1).

**Fig. 2:**
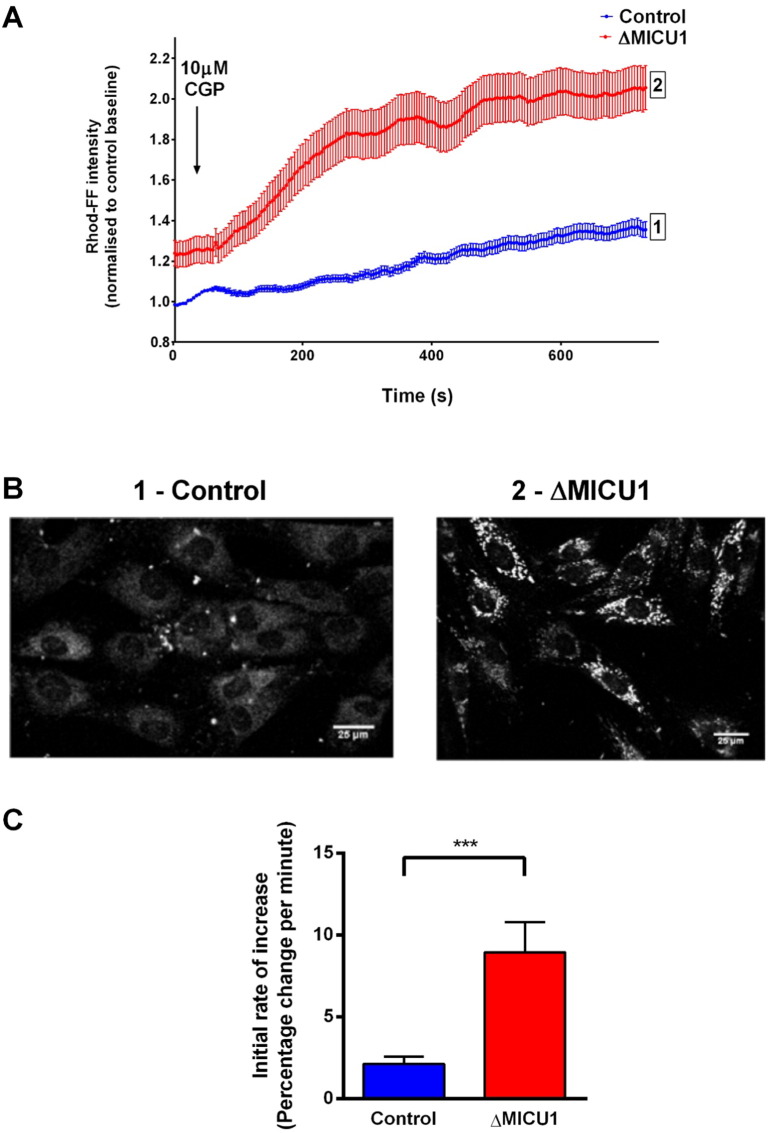
Effect of blocking the NCLX on mitochondrial Ca^2+^ uptake. (A) Representative traces of one experimental day, after normalising each time point to the resting rhod-FF intensity of the control. (B) Representative confocal images of galactose-cultured, rhod-FF loaded control [1] and ΔMICU1 [2] fibroblasts after approximately 7 mins of 10 μM CGP-37157 incubation. (C) The initial rate of increase was analysed as percentage change per minute over the first 2 min, after normalising to baseline of each cell. *n* > 100 cells from 3 independent experiments (***P ≤ 0.001).

In order to determine whether a futile Ca^2+^ cycle is active, the rate of mitochondrial Ca^2 +^ uptake was measured by imaging control and ΔMICU1 fibroblasts loaded with the low affinity mitochondrial Ca^2+^ indicator rhod-FF AM following inhibition of the NCLX using CGP-37157. Average resting rhod-FF intensity was 38.6% ± 0.9 higher in the ΔMICU1 cells than in control (*P* < 0.0001), consistent with the previous studies reporting that mitochondria are Ca^2+^ loaded at rest [44] (Fig. 2A, Supplementary Fig. S2). Immediately after exposure to CGP-37157, [Ca^2+^]_m_ started to rise in the patient cells, increasing much more slowly or not at all in control cells (Fig. 2B & C). The rate of increase of rhod-FF intensity in the patient cells was 8.9% ± 1.8 per minute compared to 2.1% ± 0.5 per minute in the control cells (Fig. 2D). It seems likely that the plateau in the rhod-FF signal represents saturation of the dye rather than true saturation of matrix [Ca^2+^]. These data confirm the activity of a continuous influx pathway in patient derived mitochondria, consistent with the presence of a futile Ca^2+^ cycle.

### 3.2 A futile Ca^2+^ cycle in ΔMICU1 cells undermines oxidative ATP generation, masked by enhanced glycolysis

**Fig. 3:**
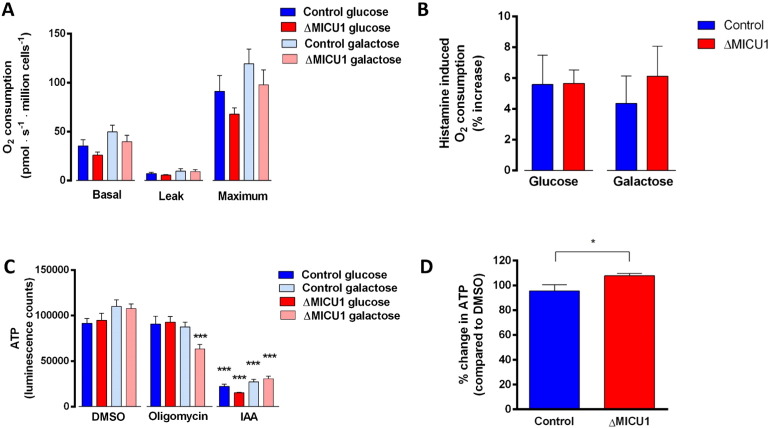
Effect of loss of function of MICU1 on oxygen consumption rate and ATP production. (A) Oxygen consumption rate of ΔMICU1 and control fibroblasts grown in glucose and galactose. (B) Oxygen consumption rate of ΔMICU1 and control fibroblasts after 10 μM histamine stimulation. Leak was measured following the addition of 2.5 μM oligomycin A and maximal respiratory capacity was measured following addition of 1 μM FCCP. *n* = 6 replicates pooled from both cell lines (2 control and 2 ΔMICU1 cell lines were measured on 3 experimental days). (C) Measurements of cellular ATP: ΔMICU1 and control fibroblasts grown in glucose or galactose were pre-treated with DMSO, 5 μM oligomycin and 1 mM IAA. (D) Cells were treated with DMSO and 10 μM CGP-37157 for 60 min. before having total ATP content quantified. Luminescence values were normalised to DMSO treatment. *n* = 6 replicates pooled from both cell lines (2 control and 2 ΔMICU1 cell lines were measured on 3 experimental days) (*P ≤ 0.05, ***P ≤ 0.001).

We previously reported that no differences were detected in the oxygen consumption rate (OCR) between patient derived cells and controls at rest or after Ca^2+^-dependent stimulation with histamine [44]. This is surprising, as one would expect a higher OCR in patient cells as a result of increased PDH activity and an increased leak reflecting the futile Ca^2+^ cycle. In an attempt to force the very glycolytic fibroblasts to adopt a more oxidative phenotype, cells were grown in galactose. However, no significant differences were seen between control and ΔMICU1 cells grown in galactose (Fig. 3 A). Furthermore, histamine stimulation did not change OCR significantly between the galactose-grown control and ΔMICU1 cells (Fig. 3 B). This could be attributed to the fibroblasts being highly glycolytic, as described previously [49]. In order to further assess the contribution of mitochondria to cellular ATP production, we measured ATP production in the patient and control fibroblasts grown in either glucose or galactose. Galactose is metabolised at a much slower rate than glucose, therefore forcing the cells to utilise glutamine and shift towards oxidative phosphorylation, resulting in increased OCR compared to cells grown in glucose [50]. Dependence of ATP generation on mitochondrial oxidative phosphorylation was significantly increased in ΔMICU1 cells compared to control cells, indicated by an increased sensitivity to oligomycin, an ATP synthase inhibitor (***P < 0.001) (Fig. 3 C). This shows that under conditions where aerobic glycolysis is reduced, ΔMICU1 cells are more reliant on mitochondrial oxidative phosphorylation to produce ATP compared to controls.

It should be noted that despite being grown in galactose, glycolysis still contributed to the bulk of ATP production in all cells, as inhibition of glycolysis in the presence of pyruvate caused a significant decrease in ATP, including application of IAA, an inhibitor of glyceraldehyde 3-phosphate dehydrogenase (***P < 0.0001) as well as pre-treating cells for an hour with 10 mM 2-deoxyglucose, a glycolysis inhibitor (***P < 0.0001) (Supplementary Fig. S3). It seems plausible that a futile Ca^2+^ cycle undermines ATP generated by oxidative phosphorylation in ΔMICU1 cells, but that this is compensated at a steady state by increased glycolytic ATP generation. We therefore measured ATP content of the cells before and after inhibition of the NCLX with CGP-37157. In ΔMICU1 cells, CGP-37157 caused a small but significant increase in ATP (108% ± 1.9) while in control cells, the inhibitor caused no significant change in ATP (95.6% ± 4.9) (*P* = 0.04) (Fig. 3 D). These findings are consistent with the proposed futile Ca^2 +^ model.

### 3.3 High resting [Ca^2+^]_m_ in ΔMICU1 cells increases pyruvate dehydrogenase activity

**Fig. 4:**
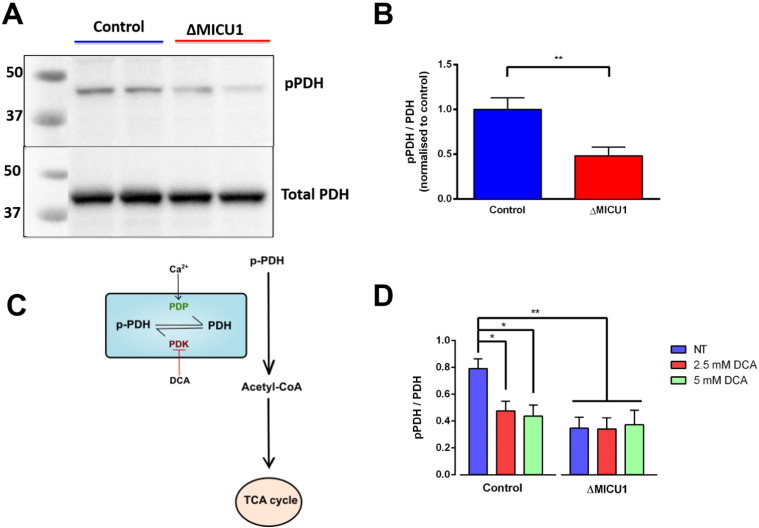
PDH is dephosphorylated in ΔMICU1 cells. (A) Whole cell lysates were immunoblotted for pPDH (PDH-E1α pS293) and total PDH (PDH-E1α). (B) The ratio of band intensities was normalised to the average control ratio on each experimental day. *n* = 8 replicates pooled from both cell lines (2 control and 2 ΔMICU1 cell lines were measured on 4 experimental days) (** *P* < 0.01). (C) Schematic diagram to demonstrate the regulation of PDH activity by Ca^2+^. DCA inhibits PDH kinase, therefore effectively activating PDH by limiting phosphorylation. Pyruvate dehydrogenase phosphatase (PDP), which increases PDH activity, is physiologically activated by Ca^2+^. (D) Cells were pre-treated with DCA before performing protein extraction. *n* = 5 for controls and *n* = 4 for patients pooled from both cell lines (1 control and 1 ΔMICU1 cell line on 4 separate days) (* *P* < 0.05, ** *P* < 0.01).

The impact of a futile mitochondrial Ca^2+^ cycle might to some extent be balanced by Ca^2+^ dependent activation of the TCA cycle due to a high resting matrix [Ca^2+^], and so the net bioenergetic effect is difficult to predict and may also vary between cell types, depending on the capacity of the TCA cycle. Increased [Ca^2+^]_m_ is expected to result in activation of the three mitochondrial dehydrogenases in the TCA cycle; isocitrate dehydrogenase, alpha-ketoglutarate dehydrogenase and pyruvate dehydrogenase [9]. The activity of PDH was assessed by measuring its phosphorylation at E1α S293, a site phosphorylated by pyruvate dehydrogenase kinase. To account for possible differences in the amount of enzyme expressed in each cell line, the ratio of pPDH intensity to total PDH intensity was quantified. In keeping with elevated resting [Ca^2+^]_m_ and enhanced Ca^2+^ activated PDP activity, the pPDH/PDH ratio was significantly reduced in ΔMICU1 cells (0.48 ± 0.097) compared to controls (1.00 ± 0.13) (*P* = 0.0064) (Fig. 4 A & B). Treating cells with dichloroacetic acid, an inhibitor of pyruvate dehydrogenase kinase, significantly reduced the phosphorylation in controls at both 2.5 mM (0.48 ± 0.07, *P* < 0.05) and 5 mM (0.44 ± 0.08, *P* < 0.05) compared to the absence of drug (0.79 ± 0.07), but had no significant effect in ΔMICU1 cells. Furthermore, the pPDH/PDH ratios in the ΔMICU1 cells for no drug treatment were comparable to phosphorylated state in the treated controls, implying that PDH is already maximally active in cells lacking MICU1 (Fig. 4 D). This agrees with our previous observations that loss of MICU1 results in increased reduced state of the NADH/NAD + pool at rest, reflecting Ca^2+^ dependent upregulation of the TCA cycle [44].

### 3.4 Loss of MICU1 expression correlates with changes in expression of EMRE

**Fig. 5:**
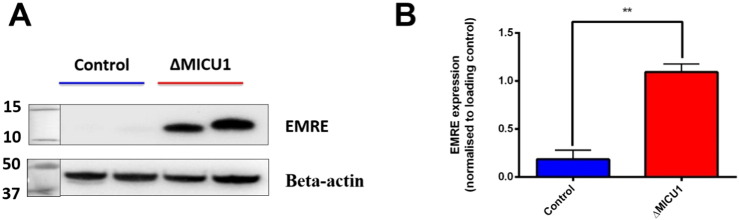
Levels of EMRE expression in control and ΔMICU1 fibroblasts (A) Whole cell lysates were immunoblotted for EMRE and beta-actin (B)The ratio of band intensities was normalised to beta actin as the loading control on each experimental day. *n* = 8 replicates (2 control and 2 ΔMICU1 cell lines were measured on 4 experimental days) (2 control and 2 ΔMICU1 cell lines on 4 separate days) (** *P* < 0.01).

The stability of the MCU complex has been linked to interactions between the different gatekeepers such as MICU1, MICU2 and EMRE, most notably in the direct correlation between MICU1 and MICU2 protein expression where loss of MICU1 leads to a downregulation in MICU2 levels. Previously, we have shown that loss of MICU1 expression did not influence the levels of MCU protein [44]. Furthermore, electron chain complex activities were not altered [44]. The role of EMRE in the formation of the complex is not fully elucidated, but has been shown to be important in interactions between the MICU1-MICU2 and MCU complex. The ΔMICU1 cells showed a significantly higher expression of EMRE compared to the controls (controls: 0.18 ± 0.095, ΔMICU1: 1.09 ± 0.085), consistent with data from the MICU1 KO mouse (Fig. 5).

### 3.5 Mitochondrial fragmentation observed in patient cells lacking MICU1 is a result of increased mitochondrial fission

**Fig. 6:**
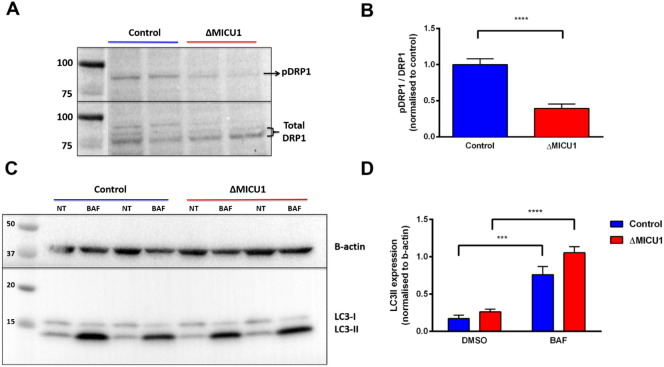
Immunoblots from whole cell lysates from galactose-cultured control and ΔMICU1 cells. (A) Immunoblot of DRP1 (S637) and total DRP1. (B) The ratio of band intensities was normalised to the average control ratio on each experimental day. *n* = 8 replicates pooled from both cell lines (2 control and 2 ΔMICU1 cell lines were measured on 4 experimental days). (C) Immunoblots for LC3. Cells were treated with 1 μL/mL DMSO (NT) or 100 nM bafilomycin A1 (BAF) for 5 h prior to protein extraction. (D) Intensity of the LC3-II band at 14 kDa was normalised to b-actin loading control. *n* = 6 replicates pooled from both cell lines (2 control and 2 ΔMICU1 cell lines were measured on 3 experimental days) (*** *P* < 0.001, **** *P* < 0.0001).

We have previously reported that mitochondria were fragmented in the ΔMICU1 patient cells compared to the controls [44]. In order to investigate the upstream and downstream pathways regulating the fragmentation of the mitochondrial network in the patient fibroblasts, we first assessed the phosphorylation status of S637 (inhibitory phosphorylation site) of DRP1. Dynamin-related protein 1 (DRP1) is a cytosolic GTPase, which is recruited to the mitochondria and drives fission [51]. The ratio of total pDRP1 intensity to total DRP1 intensity was significantly reduced in the patient cells (0.39 ± 0.06) when compared to controls (1.00 ± 0.08) (*P* < 0.0001) (Fig. 6 A & B). This suggests that DRP1 is more active in the patient cells, therefore upregulating mitochondrial fission.

Fragmentation of the mitochondrial network could result from impaired removal of fragmented mitochondria by autophagy [52]. To assess autophagic flux under basal conditions, the amount of LC3-II was measured in DMSO and bafilomycin treated control and ΔMICU1 fibroblasts. Bafilomycin, an inhibitor of the vacuolar H^+^ ATPase which therefore prevents fusion of autophagosomes with lysosomes and thus prevents their degradation, significantly increased the level of LC3-II in both control (*P* = 0.001) and ΔMICU1 cells (*P* < 0.0001). There was no significant difference (*P* = 0.827) in basal LC3-II levels between control (0.17 ± 0.02) and ΔMICU1 cells (0.26 ± 0.01), nor was there a significant difference between control (0.76 ± 0.05) and ΔMICU1 cells (1.05 ± 0.03) (*P* = 0.051) in LC3-II levels following exposure to bafilomycin (Fig. 6 C&D).

## 4 Discussion

The sigmoid dependence of mitochondrial Ca^2+^ uptake on [Ca^2+^]_c_ was established over 50 years ago, but only very recently have we begun to understand how this relationship is defined through the regulation of MCU opening by MICU1 and MICU2, while the disease phenotype caused by its failure in children with MICU1 mutations highlights its functional importance. Our own and other recently published data suggest that the MICU1/MICU2 complex prevent mitochondrial Ca^2 +^ uptake at resting cytosolic [Ca^2 +^], acts to discriminate ‘significant’ Ca^2+^ signals from noise and protects mitochondria from unwanted consequences of increased mitochondrial Ca^2+^ uptake at rest [34]. Given the Ca^2+^ dependent upregulation of the TCA cycle that accompanies a rise in matrix [Ca^2+^], that should increase oxidative phosphorylation, the observation that a futile Ca^2+^ cycle consequent on loss of MICU1 function is sufficient to undermine oxidative phosphorylation highlights the energetic costs of mitochondrial Ca^2+^ signalling, an aspect that has been somewhat neglected. It seems plausible that the relative contributions of these two opposing mechanisms may differ between cells and tissues depending on the capacity of the TCA cycle, the capacity of the respiratory chain to respond, and the activity of the NCLX may all differ between cell types and so define the tissues affected in the patients with MICU1 mutations.

The presence of a futile Ca^2 +^ cycle was confirmed by the simple experiment of blocking the efflux pathway, using CGP37157 to inhibit the NCLX. This revealed the continuous constitutive Ca^2+^ influx into the mitochondria in patient derived cells, as the matrix Ca^2+^ concentration rose immediately after inhibition of the extrusion pathway. To our knowledge, this is the first time that a futile mitochondrial Ca^2+^ cycle has been directly demonstrated in a human disease, although the mechanism has been widely considered [53], [54]. Differences in pPDH/PDH ratio between controls and patients confirmed the increase in resting matrix Ca^2+^ in the patient derived cells, and has been described in the other cohort of patients lacking *MICU1* [45], contrasting with the effect of MCU KO on PDH phosphorylation [28]. Treatment with DCA, an activator of PDH, suggested that PDH was already maximally activated in patients compared to controls, as DCA did not decrease the inhibitory phosphorylated state of the enzyme.

Understanding the impact of MICU1 loss on ATP homeostasis in the fibroblasts is difficult as the cells show a strong dependence on glycolysis for ATP production. Growing the cells in galactose, forced the cells to become more reliant on oxidative phosphorylation and so aids the detection of defects in mitochondrial respiration [50]. The patient cells showed a stronger dependence on oxidative phosphorylation for ATP production than controls. That this is undermined significantly by the futile Ca^2+^ cycle was confirmed by the small but significant increase in ATP seen in the patient cells following inhibition of the cycle using CGP-37157, while in the control cells, if anything, the drug caused a small decrease in ATP. It seems likely that the loss of the gatekeeping function in mitochondrial Ca^2+^ uptake would compromise the metabolic response to increased energy demand in the ΔMICU1 cells, with a particular impact on neurons and muscle, which show a very big dynamic range of metabolic activity.

The stoichiometry and stability of the MCU complex is currently still being investigated. Increased EMRE levels in the cells lacking MICU1 might indicate a relationship between MICU1 and EMRE expression. Our data is consistent with the MICU1 KO mouse, where EMRE expression was directly correlated with severity of symptoms [47]. Whether loss of MICU1 affects protein expression, stability of the EMRE subunits or influences proteolytic pathways that prevent EMRE turnover as seen in Konig et al., is yet to be seen.

Perturbations in mitochondrial fusion-fission dynamics have been associated with disease, most notably in neurodegenerative diseases such as Charcot–Marie–Tooth Disease and Dominant Optic Atrophy [55]. Cytosolic Ca^2+^ has been known to play a role in mitochondrial fragmentation in some studies [56], [57]. We found that phosphorylation at the inhibitory site of DRP1 was decreased in ΔMICU1 cells, indicating increased DRP1 activity. We found no change in autophagic flux in association with the mutation. Modulation of fission by altered mitochondrial Ca^2+^ homeostasis has been described in response to inhibition of the MCU, which caused downregulation of DRP1 expression [58], [59]. Additional cytosolic factors orchestrate the role of cytosolic DRP1 in mitochondrial fission. Calcineurin is activated by a rise in [Ca^2+^]_c_ and dephosphorylates DRP1, linking mitochondrial fragmentation with [Ca^2+^] signalling, but it isn't entirely clear how this relates to changes in matrix Ca^2+^ handling. If anything, the increased rate of Ca^2+^ uptake into mitochondria associated with MICU1 mutations are accompanied by a *decrease* in cytosolic Ca^2+^, and so at present we cannot readily explain the mitochondrial morphological phenotype in the fibroblasts [44].

In conclusion, this study highlights a mechanism leading to a bioenergetics deficit, which could explain the pathological phenotype that the *MICU1* patients present with, and illuminates further avenues of investigation in order to delineate the underlying pathways that might explain tissue specific responses to this loss-of-function mutation.

## Transparency Document

Transparency document Image 1

## Appendix A Supplementary data

Supplementary material Image 2
