# Supplementary material for: Pathological consequences of MICU1 mutations on mitochondrial calcium signalling and bioenergetics
Source: Biochim Biophys Acta. 2017 Jun;1864(6):1009–17. doi: 10.1016/j.bbamcr.2017.01.015 (PMC5424885; doi:10.1016/j.bbamcr.2017.01.015)
Supplement: Supplementary file 2 — Supplementary material [file mmc2.pdf]

10 Supplementary Information

10.1 Supplementary Methods: Rates of cellular ROS generation

Cells were imaged in the presence of 5µM dihydroethidium (DHE) (Molecular Probes) on a Zeiss 510 UV-VIS CSLM using a 40x oil objective and a 37°C stage. Blue fluorescence of the reduced form of DHE was excited at 351nm and emission detected between 435-485nm. Oxidised DHE was excited at 543nm and emission detected >560nm. After background subtraction, a ratio of oxidised / reduced DHE fluorescence was calculated and the rate of change in this ratio was interpreted as the rate of ROS production. The first minute of measurements were excluded to allow for loading of the dye.

10.2 Supplementary Figures

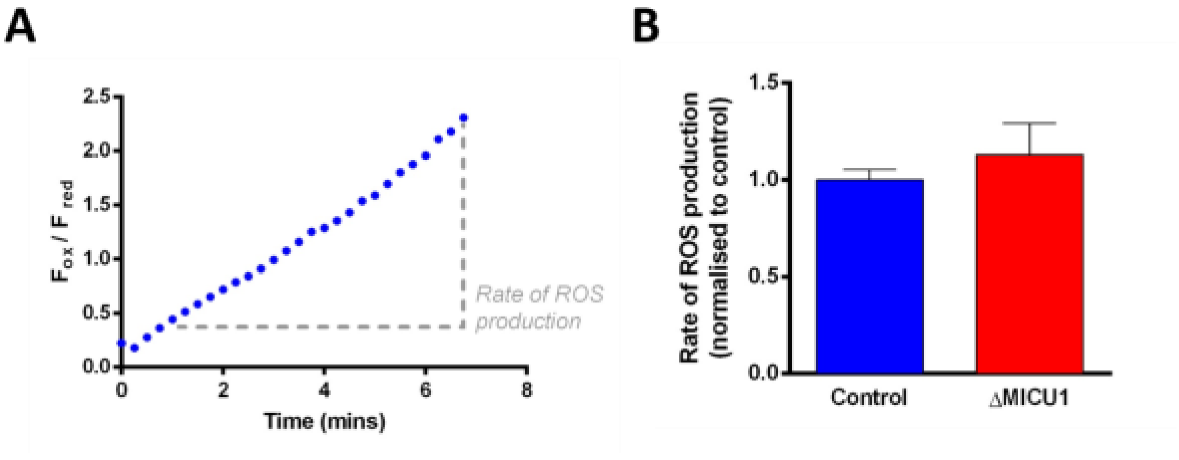

Figure S 1. Effect of MICU1 loss on cellular ROS production.
(A). Representative trace of DHE oxidation in cells. F_{ox} indicates the blue fluorescence intensity emitted by oxidated DHE, while F_{red} indicates the red fluorescence intensity emitted by reduced DHE. Rate of ROS production was calculated as the change in F_{ox} / F_{red} over time (shown in grey). Average rates of ROS production were normalised to average control rate on each experimental day.
(B). n=6 replicates pooled from both cell lines (2 control and 2 ΔMICU1 cell lines were measured on 3 experimental days). At least 8 cells were measured per cell line per day. Therefore, values shown are mean of means for each replicate. Errors bars show SEM.

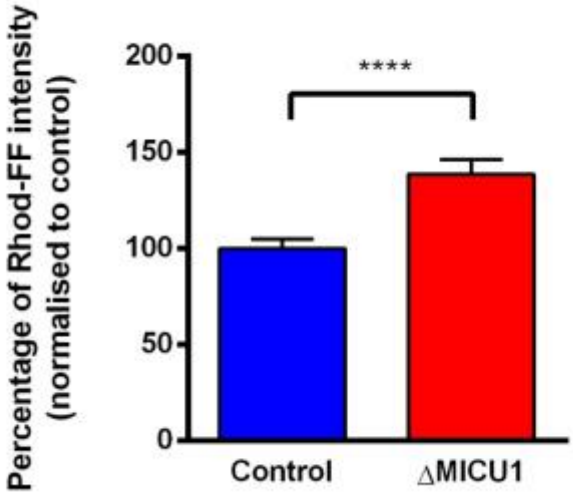

Figure S 2. Effect of MICU1 loss on resting [Ca^{2+}]$_m$
Resting rhod-FF intensity was calculated as the average fluorescence of each cell over the first 30 secs and normalised to the control mean.
n>100 cells from 3 independent experiments
(** P ≤ 0.0001)**

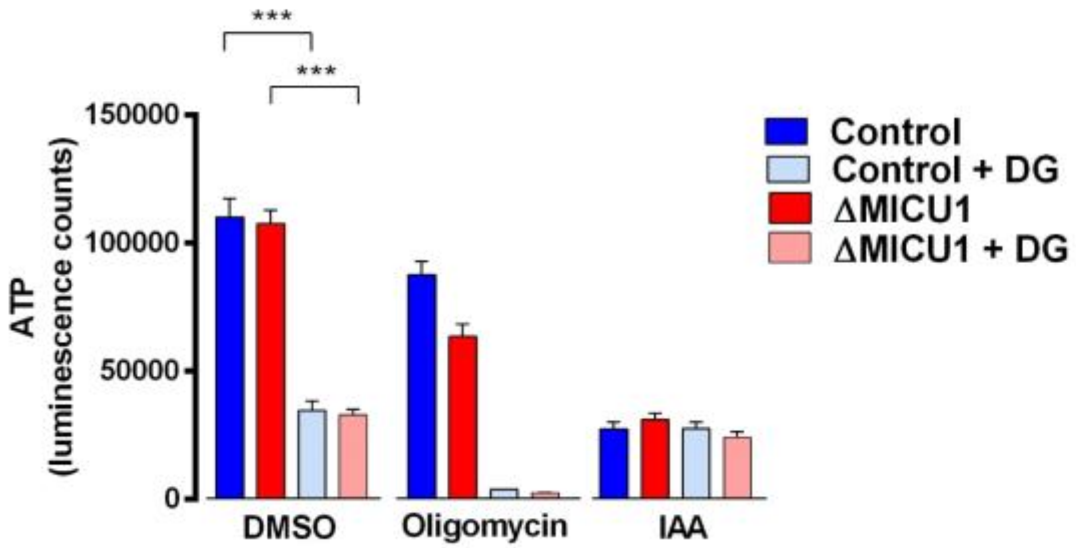

Figure S 3. Effect of 10mM 2-deoxyglucose treatment on galactose-grown cells. The effect of oligomycin on the treated cells indicates that they are still very glycolytic. As before, ΔMICU1 cells were more sensitive to oligomycin compared to the controls.
n=6 replicates pooled from both cell lines (2 control and 2 ΔMICU1 cell lines were measured on 3 experimental days)
(* P ≤ 0.001)**
